# Underwater Acoustic Impulsive Noise Monitoring in Port Facilities: Case Study of the Port of Cartagena†

**DOI:** 10.3390/s19214672

**Published:** 2019-10-28

**Authors:** Ivan Felis Enguix, Marta Sánchez Egea, Antonio Guerrero González, David Arenas Serrano

**Affiliations:** 1 Naval Technology Center (CTN), 30320 Fuente Álamo, Murcia, Spain; martasanchez@ctnaval.com (M.S.E.); davidarenas@ctnaval.com (D.A.S.); 2 Department of Automation and Systems Engineering, Technical University of Cartagena, 30202 Cartagena, Murcia, Spain; antonio.guerrero@upct.es

**Keywords:** underwater noise monitoring, impulsive underwater noise, continuous underwater noise, marine contamination, hydrophone, marine strategy framework directive, ASV

## Abstract

Recording underwater impulsive noise data is an important aspect of mitigating its environmental impact and improving maritime environmental management systems. This paper describes the method used and results of the spatial monitoring of both the baseline noise level and the impulsive noise sources in the Port of Cartagena. An autonomous vessel was equipped with a smart digital hydrophone with a working frequency range between 10 and 200 kHz and a received voltage response (RVR) of, approximately, −170 dB re 1V/µPa. A GIS map was drawn up with the spatiotemporal distribution of the basal sound pressure levels by coupling the acoustic data with the vessel’s GPS positions to identify the sources of the impulsive noise of interest and their temporal characteristics. The loading of cargo containers was identified as the main source of impulse noise. This study is the first of a series designed to obtain accurate information on underwater noise pollution and its potential impact on biodiversity in the Port of Cartagena.

## 1. Introduction

Intensive port activity is sending an ever-growing amount of artificial underwater sound into the sea, mainly due to maritime traffic. This situation has been well documented for many years, especially by the world’s navies [1]. Continuous underwater maritime transport noise has increased in recent decades [~3 dB per decade] [2]. In [3], recordings made in the 1960s were compared with others from the 1990s on the continental slope off Point South, California, showing how underwater noise related to maritime traffic has increased by up to 10 dB at frequencies of 20 to 300 Hz.

Underwater noise affects marine organisms in many ways [4], disrupts acoustic communications and auditory systems, alters behavior, and changes population distribution and abundance. Underwater noise generates physiological stress in marine organisms, stimulating nervous stress, increasing the metabolism, and reducing their immunity. These sounds affect not only aquatic mammals but also fish, amphibians, reptiles, and even invertebrates [5,6,7]. A study conducted in the Sado estuary in Setúbal, Portugal, one of the country’s largest ports, revealed changes in the behavior of bottlenose dolphins. During daylight hours, they spent less time in areas with the highest underwater noise levels [8].

Given this information on the adverse effects of noise pollution, regulatory bodies increasingly require an assessment of the acoustic underwater noise in a marine habitat before a construction project. In a review of noise modeling processes in [9], the factors that affect underwater noise generation and basic aspects of environmental impact assessment (EIAs) are highlighted.

Human activities at sea generate different types of noise that vary in frequency and intensity and can be stationary and low frequency or impulsive and high intensity. The latter are produced in pile driving, underwater blasting, seismic exploration, or sounding activities, among others. Ports can also be subjected to impulsive noise due to activities such as pumping systems, air conditioning, civil engineering works in ports, and loading containers. In [10], recordings of underwater noises were made in the port of Fremantle Inner Harbor for several months. Subsequent analyses of these data detected underwater noise from various sources, these being vessel traffic, vehicles passing over a nearby bridge, machinery noise from regular operations in the port, and vibratory or impact pile driving recorded during during wharf construction.

Impulsive noises have adverse effects on marine life. In [11], which measured and modeled pile driving noise from a large number of points around Australia, an acoustic propagation model was proposed that allowed these results to be extrapolated to other locations. Another example, [12], on pile driving in the Ligurian Sea, found that fin whales (*Balaenoptera physalus*) avoided areas with impulsive noise. Another study, [13]. also analyzed whales’ reactions to underwater impulsive noise; even 72 h after an airgun shooting period in the Alboran basin, fewer whale and received song levels were detected.

The aim of this study was to perform an acoustic characterization of impulsive underwater noise at the Port of Cartagena. This characterization was carried out according to the methodological criteria for the implementation of the Marine Strategy Framework Directive [14], including:

- Location and identification of impulsive noise emission sources.
- Quantification of background levels and continuous noise.
- Quantification of impulsive noise levels.
- Propagation of the values obtained beyond the domain of the port.

For this, a series of underwater acoustic measurements were taken in the bay, as described in Section 2.

## 2. Identification of Activities Potentially Generating Impulsive Noise in Port Facilities

In accordance with the methodological guidelines published by the European Commission [15,16,17], the main sources of impulsive sound are

- Pile driving [18]—installing piles requires the use of a large mechanical hammer to drill the seabed. Sound levels of mechanical driving can reach up to 260 dB re 1 μPa re 1 m in the absence of noise reduction measures. In [18], the impact of sound measurements had a duration of 0.2 s, the signals showed peak energy at 160 Hz, and also had significant energy up to and beyond 100 kHz.
- Explosions [19]—explosives are used as an alternative to heavy machinery to break up hard substrates, for example, in the construction of docks or deepening ports. In [19], the noise measured from the explosions had an sound exposure level (SEL) of ≥ 190 dB re 1 μPa^2^·s.
- Compressed air guns [20]—compressed air cannons are commonly used in seismic surveys to determine the geological structures of the seabed. These guns send shots of compressed air towards the seabed. The sound bounces off the bed and returns to the surface, where it is recorded by hydrophones. In [20], a 0.33 L Bolt PAR 600B air gun was used at 1500 psi (5 m depth). In these operating conditions it had a source level of 192 dB re 1 μPa^2^·s at 1 m.
- Sonar [21]—used especially by naval vessels to detect underwater objects. Simple sonar systems direct sound (short pulses) in one direction, although there are more complex systems that can emit beams of sound in multiple directions. Examples of acoustic characteristic of sound sources of this type include: multi-beam sonar with center frequency 15.5 kHz, sound pressure level (SPL): 237 dB re 1 μPa at 1 m (RMS) with omnidirectional beam; sub-bottom profiler with center frequency 3. 5 kHz, SPL: 204dB re: 1uPa at 1 m (RMS); or AN/SQS 56 sonar with center frequency of 6.8 kHz; 7.5 kHz; 8.2 kHz, SPL: 223 dB re 1 μP at 1 m (RMS) horizontal beam.
- Echosounders [21]—use sound production to locate the depth of the marine environment or schools of fish. They are used on most vessels, on fishing boats, and also on a large number of pleasure craft. For example, the SimradEK 500 Scientific echo sounder has a frequency of 38 kHz, 3 dB beam width 6.9 deg, peak transmit power 2000 W, pulse duration 1 ms and a 3.8 kHz bandwidth.
- Acoustic deterrent devices [22]—are used to alert or frighten away marine animals with sounds to avoid their interfering with fishing gear or aquaculture cages. An example of this type of device is the Ferranti-Thomson MK2 4X, which has a source level (re 1 μPa) of about 200 dB at 25 kHz. Other commercial devices have similar characteristics.

Some of these sources are regularly present in the port area, such as bathymetries and construction projects. In addition to the sources mentioned above, this study also considered the possibility of analyzing other routine activities, such as the loading and unloading of containers to analyze their potential impulsive noise production.

## 3. Materials and Methods

### 3.1. Characterization of the Submarine Bottom

**Figure 1:**
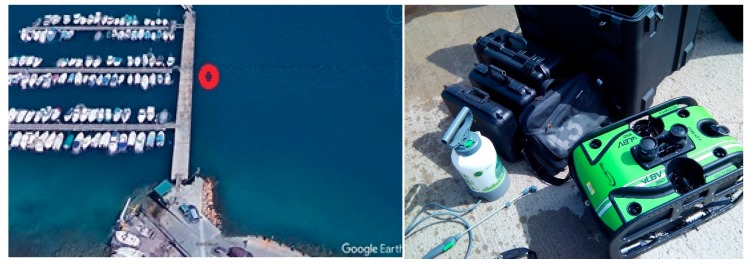
Deployment point inside the port (**left**) and one of the robotic equipment used (**right**).

The characterization of the properties of the water column and the seabed is fundamental for the implementation of acoustic propagation models. For this purpose, two types of deployment were made:

1. Visual inspections were made with underwater ROVs (remote operated vehicles) in order to determine the type of seabed in Cartagena Bay. For this, the following vehicles were used: the Deep Trecker DTG2 and Seabotix VLBV950. Figure 1 shows the location of the deployment point inside the port (left) and one of the robots used (right). Photographic and video reports were obtained.
2. A bathymetric survey was carried out to profile the seabed by means of an echo sounder using a Seaking SBP (parametric sub-bottom profiler). This sonar emits two frequencies: low frequency 20 kHz (parametric) with a beam width of 4.5º, and high frequency at 200 kHz with a beam width of 4º. The pulse width is 100 μs. When producing a 20 kHz pulse, the device is able to penetrate the seabed and highlight structural differences that conventional echo sounders cannot detect.

**Figure 2:**
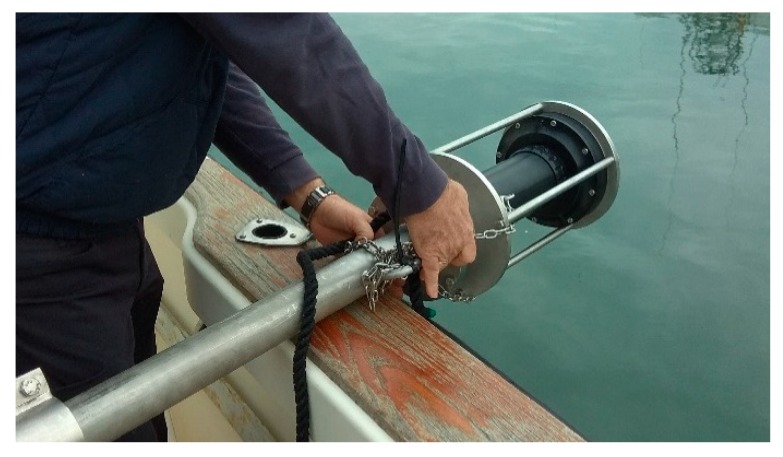
Tooling for joining the echosounder to the side of the boat.

For the measurement campaign, a special fastening element was designed and built for the SBP sonar. This clamping element allowed the sonar to be kept at the required depth without movement and vibrations. Figure 2 shows the coupling of the support to the sonar on the boat.

**Figure 3:**
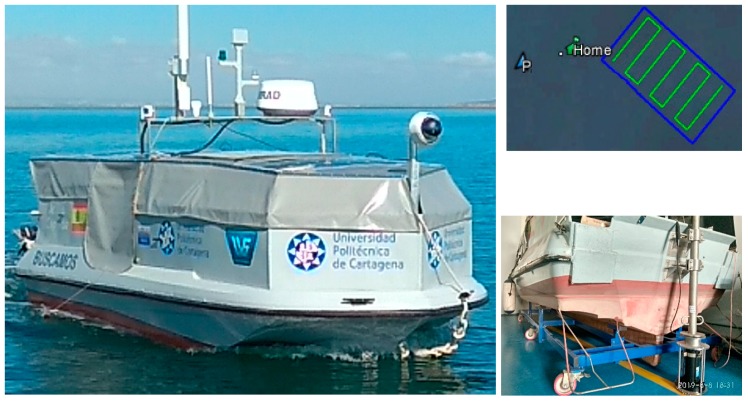
ASV with SBP (parametric sub-bottom profiler) (**left**), coupling of support to the boat (**bottom right**), ASV course definition (**top right**).

The bathymetry survey was performed by an autonomous surface vehicle ASV (see Figure 3). The ASV hull was 5.10 m long and 1.97 m wide. The displacement of the vessel under normal operating conditions was approximately 1000 kg, with an average draft of 0.325 m. The ASV was steered by two independent outboard propellers anchored to the transom. Further information on the ASV can be found in [23].

The SBP was coupled to the stern of the ASV, as shown in Figure 3 (bottom right). The sub-bottom system received the GPS positions used to reference all the sub-bottom information obtained. The sub-bottom system was connected to the hardware and software architecture of the ASV control system and synchronized the data with the position and course of the vessel. This system was capable of obtaining accurate and high-resolution sub-bottom information of the study area.

### 3.2. Characterization of Underwater Noise

Two types of measurements were carried out for the monitoring of underwater sound in the Port of Cartagena:

- Type 1: Recordings at different points around the port perimeter by manual deployment of a hydrophone.
- Type 2: Recordings in the interior of the port as well as in the access zone outside the port infrastructure by submerging a hydrophone from a boat.

**Figure 4:**
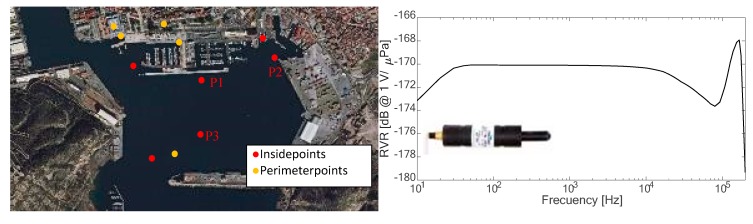
(**left**) Location of all the measurements points. (**right**) Received voltage response (RVR) of the hydrophone used.

The GPS position of each measuring point was also recorded for the subsequent spatial processing of the noise data. Figure 4 shows the position of the points.

All the measurements were made by an IcListen HF smart hydrophone. This transducer has a sensitivity of approximately −170 dB re 1V/μPa with a bandwidth between 10 Hz and 98 kHz. The device acquires signal with a sampling frequency of 192 kS/s and its bit-depth is 24 bits. Figure 4 (right) shows its received voltage response (RVR) sensibility according to the frequency band.

In both types of measurements, the hydrophone was submerged 6 meters in a series of 3 min recordings of events due to:

- Continuous sound—different types of boats passing close to the measurement points to be contrasted with the background noise. These recordings helped us to characterize the sources of continuous noise with levels above that of the background noise.
- Impulsive sound—due to boat sonars and especially to loading containers onto cargo ships. An initial estimate of potential impulsive noise inherent to the activity in the port was made from the spectrum, duration, and periodicity of these events. The recorded levels were also the basis for the further development of the numerical propagation model.

### 3.3. Processing of Undewater Noise

The signals were processed to obtain information on the frequency domain as well as the time-frequency domain.

For the frequency information, each 5 min recording was filtered in each third octave band with center frequencies from 10 Hz to 100 kHz. The average and standard deviation of the sound pressure level (SPL) was calculated from the signals obtained by windowing the filtered signals in 10 s sections. The average was calculated from $p_{rms}=\frac{1}{N}\sum_{i=1}^{N}p_{i,rms}$ and the standard deviation from $\delta p_{rms}=\sqrt{\frac{1}{N}\sum_{i=1}^{N}{\left(p_{i,rms}-p_{rms}\right)}^{2}}$, where $p_{i,rms}$ is the root mean square pressure of each windowed signal. From the resulting values, the SPL was calculated according to its usual definition [24], $SPL=20\log\left(p_{rms}/p_{ref}\right)$ and $\delta SPL=20\log\left(\delta p_{rms}/p_{ref}\right)$, where $p_{ref}=1\;\mu Pa$. Although the variability of the SPL may change according to the window used, this calculation provided an approximation of the error involved in quantifying the SPL.

A spectrogram with a rectangular window able to identify the variations of SPL with respect to the background was used for the time-frequency domain.

### 3.4. Underwater Sound Propagation Model

Several mathematical models can be used to study the underwater acoustic propagation (parabolic equation model, the based on normal modes, the spectral integration model, among others). In each case, the implementation of these mathematical models required several parameters whose exact value was not always known, which could lead to unreliable results. In order to simplify this problem, several semi-empirical models can be found that distinguish different types of analytical propagation phenomena [25].

The depth of water in the Port of Cartagena (~12 m) is such that there are multiple rebounds of the signal between the surface and the seabed, which leads to a considerable level of interaction between the propagated and reflected acoustic signals. This interaction is quite complex, as it is necessary to take into account the type of seabed, type of sediment, how it is distributed, and possible depth variations, among others. This behavior means that there is a shallow-water propagation channel.

The transmission loss of sound propagation in shallow water depends upon many natural variables of the sea surface, water medium, and bottom. Because of its sensitivity to these variables, the transmission loss in shallow water is only approximately predictable in the absence of precise values for the variables. The semi-empirical Marsh–Schulkin expressions based on the Colossus models are useful for rough prediction purposes [24,26]. This model can be used for simulations between 0.1 and 10 kHz. From this model, the transmission losses are obtained according to the equations given in:

(1) $$TL=\{\begin{array}{cc} 20\log\left(R\right)+\alpha R+60-k_{L} & R<H\\ 15\log\left(R\right)+\alpha R+\alpha_{T}\left(\frac{R}{H}-1\right)+5\log\left(H\right)+60-k_{L} & H<R<8H\\ 10\log\left(R\right)+\alpha R+\alpha_{T}\cdot \left(\frac{R}{H}-1\right)+10\log\left(H\right)+64.5-k_{L} & R>8H \end{array}$$

where R is the distance from the source; α is the absorption coefficient of the water; $k_{L}$ is a parameter called *near-field1 anomaly*, which measures the gain due to rebounds between the background and the surface; $\alpha_{T}$ is the so-called effective attenuation coefficient, which takes into account the losses due to the energy coupling between the surface and the bottom [26]; and H is the *distance of jump* or *transmission*, defined as that maximum distance at which a ray contacts the surface or the background, whose shape depends on the depth of the water column, D, and is given by the following equation:

(2) $$H=\sqrt{\frac{L+D}{3}}$$

In the study, the model was applied to the port considering 1 kHz, a calm sea, and a sandy bottom:$\;k_{L}$ = 6 dB/bounce and $\alpha_{T}$ = 1.8 dB/bounce.

It should be noted that this model was simplified and did not account for a large number of effects that should be taken into account in a more detailed propagation study. Some of these effects consider a reflection coefficient at the port perimeter and variations in depth, among others. Therefore, the numerical results of the calculations should be taken with caution. However, our intention of using a simplified propagation model here was to determine the approximate acoustic behavior of the complex propagation processes in a port environment.

## 4. Results and Discussion

### 4.1. The Submarine Seabed

**Figure 5:**
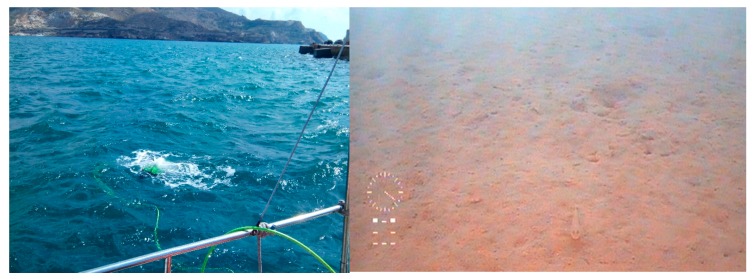
Robot on deployment (**left**) and an image of the seabed (**right**).

**Figure 6:**
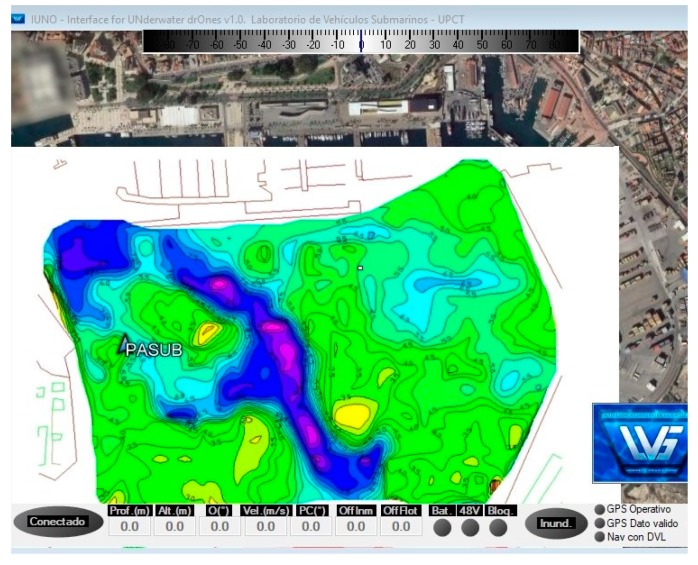
ASV control software.

The fundamental properties of the water column and the seabed were characterized by two different methods:

1. Visual inspections by an underwater ROV to obtain photographic and video reports. Figure 5 shows the robot deployed from the boat (left) and an image of the bottom at a depth of 9 m (right). The bottom was found to be soft (composed of sand, mud, small stones, and gravel) and shelved slightly with a distance from the jetty.
2. A bathymetric survey, which obtained information on the depth and types of material below the seabed, plus an updated isopach map, was carried out by a sub-bottom profiler (SBP) coupled to the stern of an ASV. The autonomous vessel was programmed to cover Cartagena Bay following parallel paths. Figure 6 shows the ASV’s control, communication, and command software, which included an initial isopach layer of the bay provided by the Cartagena Port Authority.

**Figure 7:**
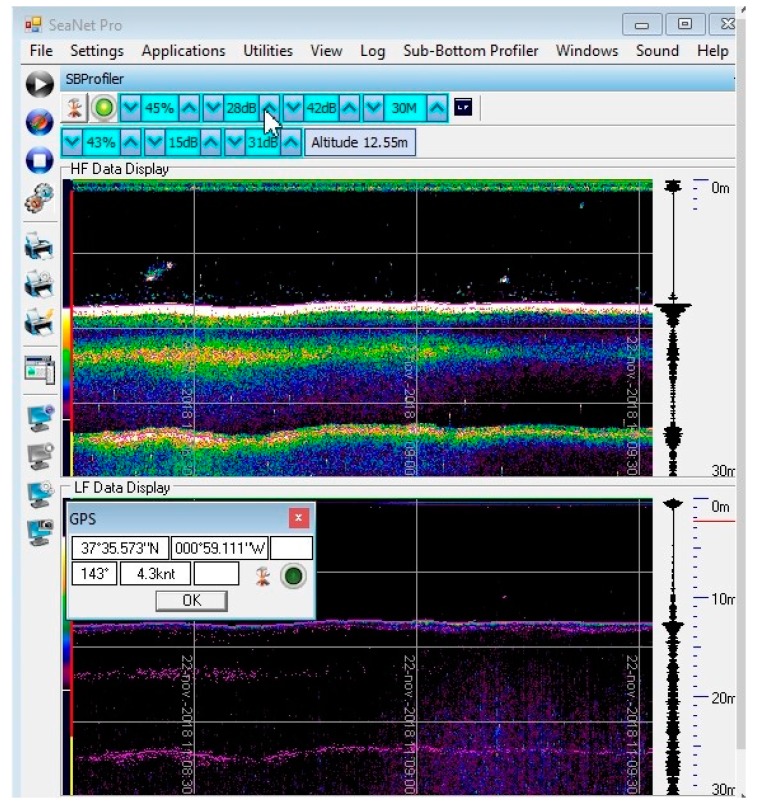
Sub-bottom profile image obtained.

A screenshot of the sub-bottom profile can be seen in Figure 7. The SBP emits a 20 kHz low frequency pulse into the seabed and highlights seismic structures and layers. The operator can view the raw high frequency profile on the screen.

The survey found a structural change at a depth of around 5 m under the seabed at certain points due to rock formations and possible sunken objects.

### 4.2. Continuous Acoustic Sound

Two types of continuous sound were differentiated to characterize the basal sound in the measurement area: (i) background sounds, recorded without the presence of marine traffic from normal port activities, waves, and other environmental factors; and (ii) continuous anthropogenic sounds from maritime traffic entering and leaving the port.

**Figure 8:**
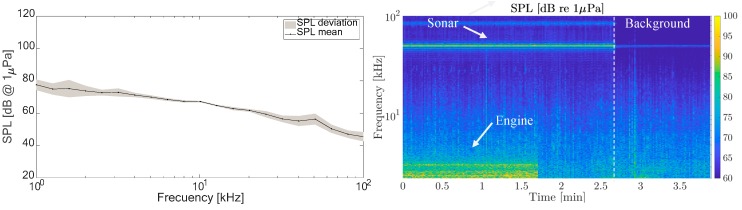
(**left**) Spectrum of background noise. (**right**) Its spectrogram at Point P1. SPL: sound pressure level.

Figure 8 gives the levels of background sound obtained and how they affect the vessels that typically operate in the port, which helped us to establish the reference level of non-impulsive sound. It shows the average SPL obtained in the octave band thirds between 1 and 100 kHz and the standard deviation calculated at all the measurement points. The average recorded SPL was 62 dB with variations of ± 18 dB. On the right is shown the changes of SPL with distance from the measurement point. Some residual sonar signals were also present. Figure 8 shows a source of the 50 kHz echosounder noise from military ships moored at a port of the bay.

**Figure 9:**
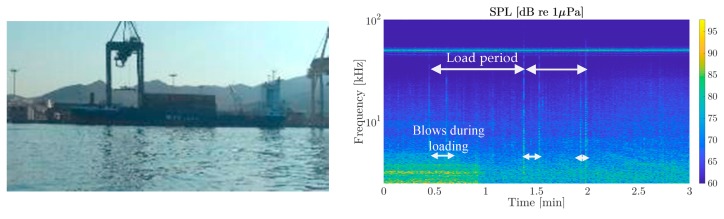
(**left**) Spectrum of continuous noise source. (**right**) Its spectrogram (Point P3).

These results gave an initial estimate of the basal sound in the port used as the basis for the study of the continuous and impulsive sound sources. However, some of the continuous sound sources, although not basal, were considered as part of the background, and not specifically impulsive sound, mainly due to passing boats. Figure 9 shows the spectrum of the influence on the background of a pilot boat passing approximately 40 m away from the measurement point.

Some vertical traces could be identified, which, as shown below, were from the nearby container loading operations.

### 4.3. Impulsive Sounds

**Figure 10:**
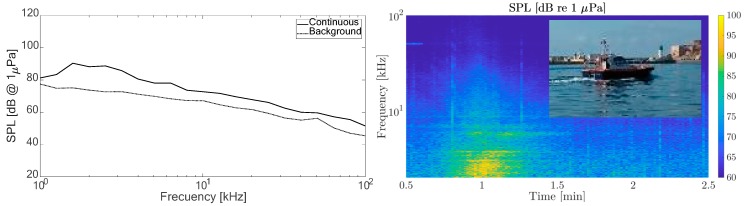
Loading a cargo ship (**left**), and spectrogram of the noise generated (**right**) at P2 in Figure 4.

During the measurement, only one source of impulsive sound was found from the process of loading sea containers onto a freighter in the eastern part of the port (Figure 10, left). A study of the spectrogram showed the levels and periodicity of these impulses (Figure 10, right). These measurements were made at approximately 50 m from the source. In the spectrogram, a 50 kHz continuous peak also appeared from naval ships docked in the port.

During the recordings, it was found that the appearance of the vertical lines in the spectrogram could be attributed to the container loading operations (especially banging noises). Some clear vertical lines can be seen in the spectrogram from this loading process. They had a considerable bandwidth (as they were pulses) with peaks of up to 85 dB at frequencies below 20 kHz. It was estimated that this impulsive source emitted around 100 dB re 1μPa re 1m.

Although this source is currently not considered as a relevant sound source [14], it may be desirable to move forward with this analysis in a context of high intensity loading and unloading operations in different ports. The possibility of including this activity as a potential impulsive noise source could also be assessed, being taken into account in noise monitoring guidelines.

### 4.4. Propagation of Underwater Noise

In these impulse noise source monitoring studies, we were interested in estimating the noise levels at various distances outside the port, as well as applying acoustic propagation models taking into account the characteristics of the water column and the soil.

Here, we applied the simplified model described in Section 3.3 to estimate the propagation range of a source with characteristics similar to the one described in Section 4.3. In addition, a ray tracing algorithm, specifically created by the Centro Tecnológico Naval y del Mar (CTN), was implemented that considered the topography of the Bay of Cartagena.

**Figure 11:**
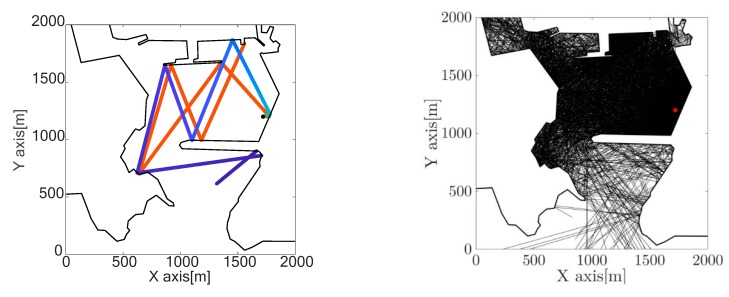
**(left**) Example of two ray traces. (**right**) All the rays traced. The red dot indicates the source.

The ray trace was generated from the position of the detected impulsive noise source within the geometry of the port. Figure 11 shows, on the left, two examples of ray trajectories up to 5 km from the source. It should be noted that one ray leaves the port while the other remains inside the limits, which affected the final propagation levels. Figure 10 shows ray traces for different emission angles.

**Figure 12:**
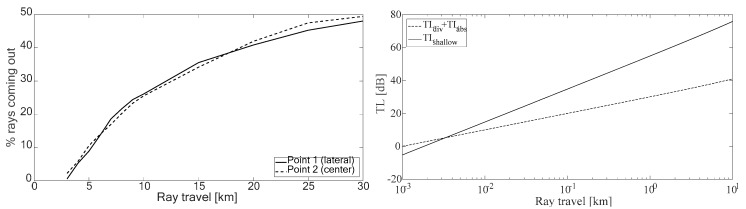
(**left**) Percentage of rays that left the port. (**right**) Comparison of transmission losses with different propagation models.

Figure 12 shows, on the left, the percentage of rays that left the port, according to their emission point. It can be seen that this position is quite independent of the number of rays that left the port, that is, the port geometry influenced whether or not they exited. Only 10% of the rays travelled up to 5 km and 20% travelled up to 7.5 km. As explained below, these distances were enough to reduce the generated impulsive noise level.

In order to detect the effect of the resulting underwater acoustic propagation along these rays, we first compared the transmission losses due only to cylindrical geometrical divergence and absorption with the transmission losses of the semi-empirical model given in Section 3.3. Figure 12 right shows the results. It can be seen that both models had the same losses within a few meters of the emission. At closer distances, the semi-empirical model presented fewer losses because, in this region, the sound propagated under a spherical divergence. For longer distances, the large number of reflections led to a sharp increase in losses that limited propagation. Specifically, for 1 kHz, there were reductions of 35 dB in 100 m and 55 dB in 1000 m, much greater than those foreseen by divergence and absorption only.

From the above results we can estimate that a source of impulsive noise with an SPL of, typically, 120 dB re 1 μPaat 1 kHz inside the port, can generate a sound wave that could travel approximately 5 km to reach the background level shown above (70 dB re 1 μPa, see Figure 12). This means the sound could travel outside the port with a level below the background noise. Nevertheless, the real losses would be smaller, as the propagation model does not consider the lost reflections, which could be of about 4 to 7 dB per collision, so that this range would decrease.

## 5. Geomaritime Representation of the Results

**Figure 13:**
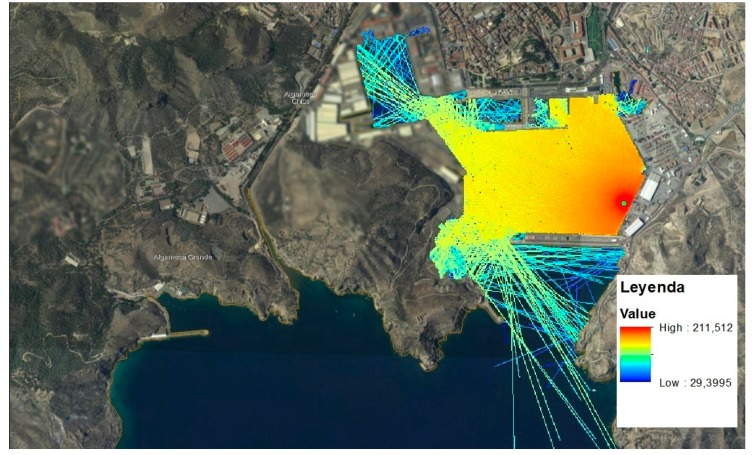
Example of submarine acoustic propagation by the web tool.

To represent the results, the CTN designed the underwater acoustic propagation model on the basis of a geographic information system (GIS) located in the study area with recorded submarine noise data. This tool can select the points where noise measurements are made and observe their temporal profile and frequency spectrum. It also allows the user to introduce a noise source and apply the simplified models of underwater acoustic propagation described above. With this, the acoustic rays propagated from the source to a previously indicated distance are obtained, plus the final sound pressure levels. Figure 13 shows the result of locating a fictitious 180 dB re μPa at a 1 kHz source next to the San Pedro dock.

## 6. Conclusions

Through this study it has been proven that

- After analyzing the background and continuous sound levels in the Port of Cartagena, it was found that impulsive sound must have a level above approximately 70 dB re 1 µPa to be detected, depending on the frequency (between 80 and 45 dB re 1 µPa in the frequency range from 10 to 100 kHz, respectively). Only one impulsive sound source was detected, and this was due to container loading operations on the east side of the port. Although this source is not usually indicated in the search for impulsive sources, these operations can be considered as a potentially impulsive noise generating activity, according to the requirements of the Marine Strategy Framework Directive.
- The analysis of the bathymetric survey of the study area was fundamental in the application of acoustic propagation models, as the type of seabed influenced the intensity of the sound, due to the reflections of the waves. Also, having reliable bathymetric information contributed to the construction of a consistent acoustic propagation model, as the accuracy of the data will also affect the accuracy of the model.
- The application of the acoustic propagation model implemented in this study, despite being approximate, provided information on the potential impact produced by the generation of impulsive noise in the vicinity of the Port of Cartagena.

In addition, this study has provided

- Measurements of impulsive sound, in accordance with the requirements of the Marine Strategy Framework Directive, which will serve to form part of the port’s underwater sound record.
- Updated and accurate data on the bathymetry of the port that can be used in subsequent studies and projects.

The results of this study can serve as a starting point for a larger scale strategy against the potential impact of underwater noise (both impulsive and continuous) in the Port of Cartagena.

To continue advancing in this regard, the following recommendations are proposed:

- Continuous monitoring of the levels of underwater sound in the Port of Cartagena, especially during activities that could potentially generate underwater noise during the periods of high sensitivity of any marine species that could be affected.
- Collection of information on marine biodiversity present in the area where activities are carried out and their surroundings. Identifying species with special sensitivity to the potential impact of submarine noise in the vicinity of the Port of Cartagena.
- Incorporating location systems for sensitive species in the port area, through, for example, passive acoustic systems.
- Adopting a set of preventive and corrective measures for activities potentially generating submarine noise, firstly related to the selection and exclusion of areas to carry out activities according to their potential impact and to minimize their environmental impact. Secondly, to control activities in seasons of special sensitivity (migrations, breeding, etc.), including the regulation of activities (navigation, works, etc.) to minimize their possible adverse effects.
